# Low Prevalence of Anti-HBc Antibody and Lack of HBV DNA Among HBsAg-Negative Blood Donors in Iran: A Cross-sectional Study and Review of Literature

**DOI:** 10.34172/aim.28579

**Published:** 2024-04-27

**Authors:** Mohammad Reza Hedayati-Moghaddam, Farahnaz Tehranian, Arman Mosavat, Rahele Miri, Sanaz Ahmadi Ghezeldasht

**Affiliations:** ^1^Blood Borne Infections Research Center, Academic Center for Education, Culture and Research (ACECR), Razavi Khorasan, Mashhad, Iran; ^2^Blood Transfusion Research Center, High Institute for Research and Education in Transfusion Medicine, Tehran, Iran; ^3^Razavi Khorasan Blood Transfusion Center, Mashhad, Iran

**Keywords:** Blood donors, Iran, Occult hepatitis B virus infection, Prevalence

## Abstract

**Background:**

Occult hepatitis B infection (OBI) refers to the presence of hepatitis B virus (HBV) DNA in the serum or liver of individuals who tested negative for HBV surface antigen (HBsAg). This study aimed to determine seropositivity for antibodies against HBV core antigen (anti-HBc) and the frequency of OBI among the HBsAg non-reactive blood donors in Mashhad, northeastern Iran.

**Methods:**

In this cross-sectional study, serum samples of HBsAg-negative blood donors were examined for anti-HBc during June and August 2018. Anti-HBc-positive samples were tested for antibodies against HBsAg (anti-HBs), and those with negative results were classified as isolated anti-HBc cases. The presence of HBV DNA in the C, S, and X gene regions was assessed by a qualitative real-time polymerase chain reaction method in all HBsAg-negative samples. OBI subjects were detected by the presence of at least one HBV genomic region.

**Results:**

Of 540 HBsAg-negative donors, 29 (5.4%; 95% confidence interval: 3.6–7.6%) showed seroreactivity for anti-HBc, of whom 18 individuals were also seropositive for anti-HBs. All donors showed negative results for all three HBV genes regardless of their serum anti-HBc status.

**Conclusion:**

Based on our findings, we suggest routine screening of Iranian blood donation volunteers for serum anti-HBc and anti-HBs but not HBV DNA.

## Introduction

 Despite extensive efforts to ensure blood safety, hepatitis B virus (HBV) infection poses a high risk, with a rate of 1 in 63,000 transfused blood units.^1^ Screening donated blood cannot eliminate the risk of post-transfusion HBV infection due to the pre-seroconversion window period, as well as the presence of occult infection in some blood donors (BDs).^2^ Occult HBV infection (OBI) refers to the presence of HBV DNA in the serum and/or liver samples without detectable HBV surface antigen (HBsAg) in the serum, along with or without the presence of antibodies against HBV core (anti-HBc) or surface (anti-HBs) antigens.^3^

 The emergence of OBI might be due to mutations in the α-determinant of the HBV *S* genomic region. Therefore, the commonly used enzyme-linked immunosorbent assay (ELISA) cannot detect the HBsAg in serum samples.^4^ In 2004, OBI was defined as HBV DNA positivity without HBsAg, with or without HBV antibodies seroreactivity, if the window period is excluded.^5^ Then, in 2008, a maximum level of 200 IU/mL for serum HBV DNA was introduced for OBI definition and those with HBV DNA > 200 IU/mL were suggested to be considered as false OBI cases.^6^ The liver cell examination for detection of replication-competence of viral DNA is the ideal method for OBI diagnosis. A molecular approach such as nested polymerase chain reaction (PCR) with amplifying at least three different regions of the HBV genome or real-time PCR assays are advised for OBI assessment. However, diagnosis is often performed using serum HBV DNA due to practical difficulties.^7^

 According to an update provided by a large number of international experts in 2018, testing more than one blood sample and the analysis of DNA extracts from at least one milliliter of serum is suggested for OBI detection.^8^ Moreover, due to the intermittent pattern of HBV DNA detection in OBI cases alongside its cost limitation, antiHBc testing can be used as an alternative method to screen occult infections and, therefore, to reduce the risk of HBV transmission.^9^ Nonetheless, it may also lead to excluding an unacceptable proportion of volunteers from blood donation due to false-positive results in areas with high rates of exposure to HBV.^10^

 Several studies have indicated that OBI is more prevalent in areas with high levels of HBV endemicity.^11^ In a systematic review of 61 studies published between 2000 and 2020 from 25 out of 31 provinces of Iran,^12^ the pooled rate of HBV infection among BDs was estimated at 0.57% (95% CI: 0.47–0.67%), with marked disparity (0.1–2.34%) across the provinces. The authors concluded that HBV prevalence has shown a declining trend over the past decades, indicating the effectiveness of the blood safety measures taken in the country. On the other hand, very high proportions of anti-HBc reactive donors (8.9–11.0%) were reported from areas of high HBV endemicity in Iran, such as the Golestan and Sistan-Baluchestan provinces.^13-15^ Moreover, we previously estimated that an average 7.9% of Iranian anti-HBc-positive BDs have molecular evidence of OBI.^16^ Also, significant OBI rates were estimated among high-risk Iranian populations such as those who inject drugs,^17^ those with hepatitis C virus or human immunodeficiency virus infections,^18^ patients with cryptogenic cirrhosis,^19^ as well as hemodialysis patients.^20^

 We previously reported rates of 1.4% and 0.33% HBsAg positivity among the general population and blood donation volunteers, respectively, in Mashhad, the capital city of Razavi Khorasan province, northeastern Iran.^21,22^ On the other hand, 8.5% of HBsAg non-reactive BDs in this city showed anti-HBc seroreactivity; however, no cases of occult hepatitis B were detected among anti-HBC-positive BDs.^23^ We aimed to determine the rate of anti-HBC seropositivity among HbsAg seronegative donors in this region and HBV DNA positivity irrespective of serum anti-HBc status.

## Materials and Methods

 During June and August 2018, a total of 540 blood samples were obtained from healthy donors who referred to Mashhad blood centers and had negative result tests for serum HBsAg and antibodies against hepatitis C and human immunodeficiency viruses. Routinely, all volunteers were assessed by a trained general practitioner, and those with a history of risky behaviors or medical conditions were excluded from blood donation. The donors with no donation history were considered first-time donors, and those who previously donated blood were defined as repeat donors.

 In the Central Lab of ACECR, Razavi Khorasan Branch, Mashhad, Iran, serum anti-HBc IgG was detected using a commercial ELISA kit (DIA.PRO, Italy, with 99.7% sensitivity and 99.5–99.8% specificity) according to the manufacturer’s instructions. Serum anti-HBs was also examined on all anti-HBc-positive samples by ELISA (DIA.PRO, Italy, with 100% sensitivity and 98.8% specificity) according to the manufacturer’s guidelines.

 In order to detect the maximum likelihood of OBI, three regions of HBV DNA (*S, C,* and *X* genes) were targeted via real-time PCR, SYBR green method (10.1128/JCM.40.11.4068–4071.2002 and 10.1097/01.EPX.0000427065.73965.c8). The viral DNA was extracted from all blood donation samples negative for HBsAg by a commercial kit (QIAamp DSP Virus Spin kit, Germany) regardless of their serum anti-HBc and anti-HBs status. The extracted DNAs were examined for HBV DNA presence using three pairs of specific primers (Table 1). HBV positive control was kindly provided by the Central Medical Diagnostic Lab of the Academic Center for Education, Culture and Research (ACECR), Razavi Khorasan Branch. Real-time PCR amplifications were performed by the RealQ Plus Master Mix Green (AMPLIQON, Denmark) via Rotorgen Q-6000 real-time PCR machine (Qiagen, Germany). The PCR amplification reactions were considered to a final volume of 25 µL, including 1 µL of each primer (10 pmol), 12.5 µL of SYBR Green Master Mix, 10 µL of DNA template, and 0.5 µL of nuclease-free water. PCR amplification for the S gene was done using a two-step PCR program, including one cycle of 95 °C for 15 minutes, then 95 °C for 15 seconds, followed by annealing and extension at 50 °C for 60 seconds, repeated for 45 cycles. Also, the C and X genes were detected as follows: one cycle of 95 °C for 15 minutes, then 95°C for 15 seconds, followed by annealing and extension at 55 °C for 60 seconds, repeated for 40 cycles. Finally, the melting curve analysis profile was 65 °C for 30 seconds and 95 °C for 30 seconds.

**Table 1:** Primer Sequences of Three Regions of the HBV Genome

**HBV Target Gene **	**Sequence (5' → 3')**	**Nucleotide Position**	**References**
Surface gene	F: AGAACATCGCATCAGGACTC R: CATAGGTATCTTGCGAAAGC	159–178 642–623	10.1128/JCM.40.11.4068–4071.2002 10.1097/01.EPX.0000427065.73965.c8
Core gene	F: CTGGGAGGAGTTGGGGGA R: GTAGAAGAATAAAGCCC	1730–1747 2503–2487	10.1128/JCM.40.11.4068–4071.2002 10.1097/01.EPX.0000427065.73965.c8
X gene	F: CTAGCCGCTTGTTTTGCTCG R: TTATGCCTACAGCCTCCTAG	1282–1301 1666–1647	10.1128/JCM.40.11.4068–4071.2002 10.1097/01.EPX.0000427065.73965.c8

 OBI cases were detected by the presence of at least one HBV genomic region (*S/C/X* genes). The detection limit was established for each HBV gene threshold (Ct/Cq value), with a positive threshold set at ≤ 38.

###  Statistical Analysis

 Using a single proportion formula, the sample size was determined to estimate the prevalence of OBI among our target population. Based on the findings of a survey conducted in Tehran Blood Transfusion Center,^24^ the frequency of HBV DNA positivity among anti-HBc-positive specimens was considered as 50% (*P* = 0.5). Considering a precision of 4% at a 95% confidence level, we estimated that 601 cases would need to be enrolled. Data were analyzed using SPSS Statistics for Windows, version 19 (IBM Corp., Armonk, N.Y., USA). Categorized variables were presented as numbers and percentages and analyzed using chi-Square and, if indicated, Fisher exact tests. Numerical variables were described using mean and standard deviation, and an independent samples t-test was conducted for analysis. Moreover, a binary logistic regression analysis was used to determine potential variables associated with anti-HBc seropositivity in the studied population. A statistical significance level of *P* < 0.05 was considered.

## Results

 From 540 HBsAg-negative BDs with a mean age of 38.4 ± 10.3 (range: 18‒62) years, nearly all participants (98.5%) were male, and most (89.6%) were categorized as repeat donors with an average of 9.5 ± 7.8 times of donation.

 A total number of 29 samples (5.4%; 95% confidence interval [CI]: 3.6–7.6%) showed seroreactivity for anti-HBc; all were male, most (72.4%) were 40 years or older, and almost all (93.1%) were repeat donors (Table 2). Multivariate analysis showed that anti-HBc seropositivity was significantly related with the participants’ age (*P* < 0.001). However, the participant’s gender (*P* = 0.99) or donation status (*P* = 0.81) were not significantly associated with a positive result for this serology test.

**Table 2:** Anti-HBc Seropositivity among Blood Donors Based on Their Age, Sex, and Donation

**Variable**	**Total, No. (%) **	**Anti-HBc Positive, No. (%) **	***P *** **Value**
Sex			
Female	8 (1.5%)	0 (0.0%)	1.0
Male	532 (98.5%)	29 (5.5%)	
Age			
< 20	8 (1.5%)	0 (0%)	< 0.001
20–29	100 (18.6%)	1 (1.0%)	
30–39	221 (41.0%)	7 (3.2%)	
40–49	120 (22.3%)	9 (7.5%)	
50–59	78 (14.5%)	10 (12.8%)	
≥ 60	12 (2.2%)	2 (16. 7%)	
Donation status			
First-time	56 (10.4%)	2 (3.6%)	0.76
Repeat	484 (89.6%)	27 (5.6%)	

 Of 28 anti-HBc reactive donors, 18 (64.3%; 95% CI: 44.1–81.4%) individuals were also seropositive for anti-HBs, of whom 12 donors (66. 7%) had antibody titers higher than 100 IU/L. Ten (35.7%; 95% CI: 18.6–55.9%) subjects with detectable anti-HBc were seronegative for anti-HBs and classified as anti-HBc only or isolated anti-HBc cases. Neither anti-HBc-positive nor anti-HBc-negative donors showed positive results for the S, C, or X viral genes. In other words, no OBI cases were found among the studied population.

 We reviewed surveys on HBsAg, anti-HBc, and HBV DNA positivity among Iranian BDs and summarized the findings in Table 3. HBsAg seroreactivity varied greatly based on the studies date and location; the highest rates were reported from the Golestan (2.2%) and Sistan-Baluchestan (2.3%) provinces in 2005, and rates as low as 0.1% were reported from the Kerman, Golestan, and Fars provinces during 2019‒2022. Regarding anti-HBc, the highest rates were reported from the Sistan-Baluchestan (20.2% in 2010), Tehran (11.5% in 2007), Markazi (11.2% in 2010), and Golestan (11.0% in 2019) provinces (Table 3). The OBI rate indicated a significant disproportionate distribution among Iranian BDs, from zero to 29.8% (one survey with an unexpectedly high frequency (50%) was omitted).

**Table 3:** Reported Prevalence of HBsAg, Anti-HBc, and HBV DNA among Iranian Blood Donors

**Province **	**First Author**	**Year**	**Sample** **Size**	**HbsAg+** **(%)**	**Study**	**Year**	**Sample** **Size**	**Anti-HBc+** **(%)**	**HBV DNA+** **(%)**
Bushehr	Esmaieli^25^	2009	20 294	0.24	—				
Chaharmahal-Bakhtiari	Doosti^26^	2009	11 200	1.79	—				
Fars	Ghavanini ^27^	2000	7964	1.07	Behzad-Behbahani^28^	2006	2000	6.55	12.21
	Emamghorashi^29^	2006	3011	0.37	Behzad-Behbahani^30^	2011	1500	5.07	15.79
	Kasraian^31^	2007	510 030	0.49					
	Kasraian^32^	2010	203 761	0.37					
	Kasraian^33^	2012	96 909	0.27					
	Azadbakht^34^	2020	1 955 162	0.14					
Ghazvin	Vahid^35^	2005	39 598	1.08	—				
Golestan	Kazeminejad^36^	2005	39 806	2.23	Tabarasad-Laleh^14^	2019	3500	11.00	0.00
	Bani Aghil^37^	2010	129 469	0.98	Bahrami^13^	2022	4313	8.90	0.00
	Bahrami^13^	2022	47 506	0.12					
Guilan	Mansour Ghanaei^38^	2008	222 505	0.45	Khamesipour^39^	2011	2041	3.82	1.28
	Taheri Azbarmi^40^	2008	49 950	0.26					
Hamadan	Rezazadeh, M.	2006	18 306	0.77	—				
	Ranjbarian, P.	2008	8468	0.47					
Hormozghan	-				Khorami^41^	2013	1000	8.30	-
Isfehan	Afzali^42^	2002	44 004	0.62	Pourazar^43^	2005	545	7.89	11.63
	Moniri^44^	2004	603	0.50					
	Masaeli^45^	2006	29 619	0.54					
	Pourazar^46^	2006	51 799	0.67					
	Ebrahimian^47^	2011	542 705	0.20					
Kerman	Arab, M.^48^	2006	15 535	1.04	Jafarzadeh^49^	2008	270	5.19	-
	Seyed Askari, SM.^50^	2015	361 559	0.23	Kazemi Arababadi^51^	2009	3700	9.51	16.19
	Mohsenizadeh^52^	2017	99 187	0.53	Delavari^53^	2011	1525	7.93	29.75
	Etminan^54^	2019	355 507	0.10					
Kermanshah & Khuzestan	—				Karimi^55^	2016	2031	4.87	0.00
Kurdistan	Maghsoudlu^56^	2018	198 136	0.29	—				
Lorestan	—				Abdi^57^	2008	1000	4.80	—
Markazi	Mahdaviani^58^	2006	11 695	0.68	Sofian^59^	2010	529	11.15	—
	Sofian^59^	2010	531	0.38					
Razavi Khorasan	Vossoughinia^60^	2010	314 154	1.16	Shahabi^23^	2009	5059	8.54	0.00
	Yazhan^61^	2016	57 507	0.30					
	Hedayati-Moghaddam^22^	2019	58 276	0.39					
Sistan-Baluchestan	Sanei Moghaddam^62^	2005	7360	2.28	Sanei Moghaddam^63^	2010	431	20.19	—
					Pazoukian^15^	2013	1500	9.60	—
Tehran	Attarchi^64^	2006	26 811	0.62	Amini Kafiabad^65^	2007	2000	11.50	0.00
	Khedmat^66^	2009	1 010 865	0.59	Vaezjalali^24^	2013	1000	8.00	50.00
	Omidkhoda^67^	2011	11 510	0.47					
	Mirrezai^68^	2014	203 099	0.23					
	Mohammadali^69^	2014	2 034 497	0.39					
Yazd	Javadzadeh Shahshahani^70^	2013	255 427	0.26	Vaziri^71^	2021	1500	4.93	0.00

## Discussion

 The current study showed that 5.4% of HBsAg non-reactive BDs in northeastern Iran are anti-HBc-seroreactive. Most provinces of Iran were classified as areas with low levels of HBV endemicity, where 5–7% of the population have been exposed to the virus and up to 2% are chronic carriers.^72^ In a systematic review of 13 studies from 12 provinces of Iran,^73^ the national rates of HBs Ag and anti-HBc Ab among the general population were estimated at 1.8% (95% CI: 1.6%, 2.1%) and 13.6% (95% CI: 12.9%, 14.3%), respectively, with marked disparity across the provinces (0.8–5.1% for HBs Ag and 4.2–36.9% for anti-HBc Ab prevalence, respectively). The authors concluded that HBV prevalence in our country is low and has shown a declining trend over the past years; however, high HBV rates were reported in some provinces.

 In 2009, Shahabi et al reported a rate of 8.5% anti-HBc seroreactivity among HBsAg non-reactive BDs in Mashhad. Compared to their report, our finding reflected a significant reduction in the rate of exposure to HBV during a ten-year period in this region.^23^ Our previous survey also showed a declining trend in the HBsAg positivity rate among BDs in northeastern Iran.^22^ Similarly, a declining trend of HBV infection among Iranian BDs has been shown in both low and high HBV prevalence regions over the past decades. Amini Kafi-abad et al^74^ reported that the overall HBsAg rates reduced from 1.8% in 1998 to 0.4% in 2007 in Iran. In the Fars province, an area with low HBV endemicity, the data declined from 0.9% to 0.3% during this period. Also, HBsAg frequency diminished from 3.7% to 1.1% in the Sistan-Baluchestan province, representing a high prevalence region. This trend could be due to improvements in donor recruitment and selection, as well as a reduction in HBV prevalence in the general population.

 Our review indicated that anti-HBc prevalence differs among Iranian BDs based on different geographical regions (Table 3). Anti-HBc could be a serological marker of past or current exposure to HBV; therefore, its prevalence might be influenced by HBV endemicity levels in different regions. In a study by Merat et al,^75^ HBsAg prevalence rates among 18–65-year-old individuals chosen in 2006 from the general population in Tehran, Hormozgan, and Golestan provinces of Iran were assessed as 2.3%, 2.7%, and 5.1%, respectively. Correspondingly, the proportion of BDs with anti-HBc seropositivity in Tehran and Hormozgan were 14.2% and 13.3%, respectively, but the rate was reported as high as 36.9% in Golestan, a province with high HBV endemicity. Likewise, our literature review revealed that the highest frequency of anti-HBc (20.2%) was reported from the Sistan-Baluchestan province (Table 3),^63^ where a considerable rate of HBsAg seroreactivity (2.3%) was reported among BDs.^62^ Similarly, the prevalence of anti-HBc seropositivity varies among BDs in the countries of the world according to HBV prevalence among the general population, with rates of 0.2% in the USA,^76^ 8.3% in Italy,^77^ 10.2% in India,^78^ and 13.5% in South Korea.^79^ In a study in the Kurdistan Region of Iraq, 0.2% of 12 185 BDs were found to be HBsAg-positive, and only 2.3% of HBsAg-negative cases showed a reactive result for antibodies to HBV core antigen.^80^ Likewise, García-Montalvo et al reported a rate of 0.2% and 4.2% positivity for HBsAg and anti-HBc, respectively, among Mexican donors.^81^

 We found that two-thirds of the donation samples negative for HBsAg but positive for anti-HBc- were also reactive for anti-HBs, and two-thirds of these (43% of all BDs) had a titer higher than 100 IU/L. Based on reports in the last decade, the rates of anti-HBs seroreactivity among anti-HBc positive BDs in different provinces of Iran were 72% to 85%.^13,15,41,55,71,82^ Similarly, some reports from Golestan and Sistan-Baluchestan, two provinces with the highest HBV frequency in the country, noticed that 46–48% of all HBsAg non-reactive and anti-HBc-positive donation volunteers had anti-HBs titer over 100 IU/L.^13,15^ However, higher rates (55–58%) were reported from other provinces, such as Yazd and Tehran.^71,82^ In developed countries such as Japan and the USA, up to 70% of people with anti-HBc positivity have anti-HBs titers above 100 IU/mL.^83^ In a study by Romanò et al, 86.7% of 2436 HBsAg-negative and anti-HBc-positive donors also had positive anti-HBs test results, of whom 63.9% revealed antibody titers > 100 IU/L.^77^ In contrast, the rate of anti-HBs positivity is much lower in low-income countries, where a high level of HBV endemicity is identified.^83^

 By investigating the three *S, C*, or *X* genomic regions, we detected no OBI cases among BDs with or without anti-HBC positivity in northeast Iran. Likewise, Shahabi et al could not find any cases of occult hepatitis B among anti-HBC positive BDs in this area.^23^ Karimi et al^55^ tested the presence of the HBV genome in serum samples from HBsAg-negative BDs in two main blood centers in western and southwestern Iran. They could not detect any HBV DNA cases either using a PCR technique with pooled specimens of 5 donations among 1932 anti-HBc-negative cases or by a single specimen real-time assay among 99 samples positive for anti-HBc.

 Our literature review indicated that the OBI rate among Iranian BDs varied from zero to 29.8% (Table 3). OBI prevalence in a specified population depends partly on HBV endemicity. Candotti et al^84^ reviewed OBI reports from Poland, Italy, Spain, and Germany and estimated only one to 51 OBI cases per 100 000 donations among the European countries with low HBV infection levels. Correspondingly, Romanò et al^77^ reported a low rate (0.33%) of HBV infection among Italian first-time BDs and identified only 12 cases (0.55%) with HBV DNA positivity among 2186 HBsAg-negative and anti-HBc-positive donors.

 In some provinces of Iran, however, OBI percentages are not fully compatible with the prevalence of HBV infection. In provinces such as Fars and Isfahan, areas with low prevalence (0.37%–0.50%) of HBsAg seropositivity,^29,32,44^ considerable rates (11.6%–15.8%) of occult hepatitis B were reported.^28,30,43^ Conversely, no definite cases of circulating HBV DNA in serum were reported among BDs in Golestan, a province with a high HBV prevalence in the country.^13,14^ It has been suggested that variation in OBI rates could be related to factors other than HBV endemicity in particular geographical regions. These variables include the power of the studies, the distribution of HBV risk factors in communities, coverage rates of HBV vaccination, and the sensitivity of serological and/or molecular HBV detection assays.^85^ OBI prevalence could be overestimated when a less sensitive method was used to diagnose HBsAg in the serum samples.^9^ Besides, our previous meta-analysis showed that the rate of occult infection was significantly different in the Iranian population based on PCR techniques (conventional, nested, or real-time PCR) used to detect circulating HBV DNA in serum.^16^ The current study used the SYBR Green Real-Time PCR method to analyze three different HBV genomic regions (*S, C, *and* X*); all donors with or without anti-HBc tested negative for the three viral genes.^10^ These diverse results highlight the challenges in diagnosing OBI and emphasize the need to identify the target population for screening.

 Our current study has some limitations. The accuracy of the homemade assay should be compared with an available standardized method. Another potential limitation could include variations in testing methods over time or the potential influence of factors not considered in the study.

## Conclusion

 Given the not-so-high prevalence (5.4%) of anti-HBc in HBsAg non-reactive BDs, it does not seem that routine screening for anti-HBc and excluding the cases with positive test results would reduce blood reserves in our region. Thus, anti-HBc testing could be added to the HBsAg test to screen all blood components to decrease the risk of post-transfusion HBV infection in Iran. On the other hand, we found that two-fifths of the anti-HBc-positive BDs had anti-HBs titer > 100 IU/L. We, therefore, suggest that an alternative strategy is designed to screen both anti-HBc and anti-HBs and to exclude only the population without a protective level of anti-HBs ( > 100 IU/L). Also, we do not propose using expensive molecular methods as a routine HBV screening tool since none of the BDs enrolled in our study, even those with anti-HBc seropositivity, had occult hepatitis.
